# Characterization of Enamel and Dentine about a White Spot Lesion: Mechanical Properties, Mineral Density, Microstructure and Molecular Composition

**DOI:** 10.3390/nano10091889

**Published:** 2020-09-21

**Authors:** Evgeniy Sadyrin, Michael Swain, Boris Mitrin, Igor Rzhepakovsky, Andrey Nikolaev, Vladimir Irkha, Diana Yogina, Nikolay Lyanguzov, Stanislav Maksyukov, Sergei Aizikovich

**Affiliations:** 1 Research and Education Center “Materials”, Don State Technical University, Gagarin Square 1, 344000 Rostov-on-Don, Russia; michael.swain@sydney.edu.au (M.S.); boris.mitrin@gmail.com (B.M.); andreynicolaev@eurosites.ru (A.N.); irkha.vladimir@gmail.com (V.I.); saizikovich@gmail.com (S.A.); 2 Biomaterials and Bioengineering department, Faculty of Dentistry, The University of Sydney, Camperdown, Sydney NSW 2006, Australia; 3 Institute of Life Sciences, North Caucasus Federal University, Pushkin Street 1, 355009 Stavropol, Russia; 78igorr@mail.ru; 4 Federal Research Centre The Southern Scientific Centre of the Russian Academy of The Sciences, Chehova Street 41, 344006 Rostov-on-Don, Russia; 5 Department of dentistry, Rostov State Medical University, Nakhichevansky Lane 29, 344022 Rostov-on-Don, Russia; dianaturbina@mail.ru (D.Y.); kafstom2.rostgmu@yandex.ru (S.M.); 6 Faculty of Physics, Southern Federal University, Bolshaya Sadovaya Street 105/42, 344090 Rostov-on-Don, Russia; n.lianguzov@mail.ru

**Keywords:** caries, enamel, dentine, nanoindentation, X-Ray microtomography, Raman spectrum analysis

## Abstract

The study focuses on in vitro tracing of some fundamental changes that emerge in teeth at the initial stage of caries development using multiple approaches. The research was conducted on a mostly sound maxillary molar tooth but with a clearly visible natural proximal white spot lesion (WSL). Values of mineral density, reduced Young’s modulus, indentation hardness and creep as well as the molecular composition and surface microstructure of the WSL and bordering dentine area were studied. The results obtained were compared to those of sound enamel and dentine on the same tooth. A decrease of mechanical properties and mineral density both for the WSL and bordering dentine was detected in comparison to the sound counterparts, as well as increase of creep for the enamel WSL. Differences in molecular composition and surface microstructure (including the indenter impressions) were found and described. WSL induces a serious change in the state of not only the visually affected enamel but also surrounding visually intact enamel and dentine in its vicinity. The results provide the basis for future studies of efficacy of minimal invasive treatments of caries.

## 1. Introduction

The study of caries from a materials and microstructural point of view, in addition to conventional bitewing X-ray techniques [1,2], provides a better understanding of the changes in the carious enamel and affected dentine. Various mechanical and spectroscopic approaches can be applied in this regard, each with its benefits and drawbacks. Hardness measurements have come a long way from the pioneering studies of over 50 years ago [3] to modern day research involving instrumented estimation of both indentation hardness and reduced Young’s modulus at the sub-micron level [4,5]. Computed X-Ray microtomography (micro-CT) is another powerful tool to study the demineralization and remineralization of teeth [6,7], it can also be applied for testing efficiency of caries treatments [8]. Raman spectroscopy enables researchers to study accurately the molecular composition of carious lesions [9]. At the same time, fluorescence spectra due to organic materials may tend to dominate the much weaker Raman signals, therefore, Raman spectroscopic studies are often limited to enamel, which contains only a small fraction of organic components [10]. Atomic force microscopy (AFM) enables quantification of the surface roughness parameters for the carious lesions thus representing another method for testing of different clinical treatments such as bleaching agents [11] or detailed observation of the enamel demineralization process [12]. This tool is mostly suitable for localized regions of interest. Scanning electron microscopy (SEM), although unsuitable to measure the parameters of the surface microgeometry, can be used to observe relatively large areas with serious height changes, such as borders of the lesions. However, additional damage to the sample can occur from exposure to vacuum or to the electron beam [13]. At the same time, the combination of different techniques provides the possibility to overcome limitations and helps to understand the processes occurring inside carious tissues [14,15].

The first clinically visible stage of the carious disease is characterized by enamel demineralization without cavitation. Such diseased enamel usually presents a near intact surface layer of 10–100 µm thickness with a subsurface porous area called the body of the lesion. The pores are formed as a consequence of partial dissolution of carbonated hydroxyapatite crystallites due to etching caused by carbohydrate metabolizing cariogenic biofilm bacteria producing organic acids [16]. Due to the significant difference of the refractive indices of the medium inside the acid-created pores of the demineralization area, a whitish opaque appearance of these lesions can be observed. This phenomenon is called a white spot lesion (WSL) [17,18]. Overall, the number of studies characterizing the fundamental changes that emerge in natural WSLs from a materials and microstructural point of view remains rather low. Huang et al. in [19] using nanoindentation and micro-CT showed excellent correlation between mineral density and elastic modulus for the enamel component of WSLs. Ko et al. [20] and Kinoshita et al. [21] successfully provided Raman spectral imaging characterization for early caries. Mapping of the mechanical properties for natural WSLs supplemented by SEM observations was conducted by Huang et al. in [22]. Metwally et al. used in vivo radiographic tracing of structure to show the stages of the remineralization process for young permanent teeth with WSLs in [23].

The present work aims to characterize the complex of properties (mineral density, reduced Young’s modulus, indentation hardness, average roughness, maximum height of roughness) and features (surface structure, molecular composition, indentation creep) of the enamel and dentine about a WSL on a single tooth using all the techniques mentioned in this section. The aim of obtaining such information is to provide the basis for future studies of efficacy of minimal invasive treatments of caries [24,25,26,27,28] and deeper analysis of these properties and features.

## 2. Materials and Methods

An extracted permanent maxillary molar was collected for orthodontic purposes from an individual (male, 21 years old) in the dental department of Rostov State Medical University clinic. Local independent ethics committee of Rostov State Medical University approved the study (statement 15/9 from 3 October 2019), the patient provided informed consent. The WSL was assessed independently by two experienced clinicians. It was located in the proximal contact zone, visually white in color with no obvious surface damage. Following extraction, the sample was kept in 1 wt. % NaClO solution for 10 min. Then the sample was stored in Hanks Balanced Salt Solution (HBSS) at 4 ºC with thymol granules (Unifarm, Slavyansk-na-Kubani, Krasnodar Region, Russia), added to prevent fungal growth and disinfection. The ratio of thymol to HBSS was 1:1000.

According to principal carious lesion classification criteria introduced in [29], the carious WSL under consideration represents a primary active incipient enamel caries lesion located on the smooth-surface of an adult patient. According to FDI World Dental Federation caries matrix [30] there are specific clinical reporting guidelines: I—sound, no obvious dentine caries, a—noncavitated enamel (+), 1—first visual change in enamel (+), where sign (+) indicates the activity of caries lesions as defined in the glossary of terms for caries [31].

We investigated four oval areas (approximately 1.4 mm × 1 mm) of the tooth:

1. natural enamel WSL;
2. dentine bordering the WSL (touching the dentine–enamel junction as close to the WSL as possible);
3. area of sound enamel on the opposite medial side of the tooth;
4. dentine bordering the area of sound enamel (touching the dentine–enamel junction and as close to the area of sound enamel of area 3 as possible).

We concentrated on the different areas of the single sample to avoid variations of experimental data attributed to noncarious factors such as age [32] and influence of the environment [33,34].

A longitudinal section through the region containing a WSL was cut using a precision saw (Isomet 4000, Buehler, Lake Bluff, IL, USA) with an abrasive SiC disc (MetAbrase, Buehler, Lake Bluff, IL, USA). The pulp chamber was cleaned of remnants of soft tissues. The cut surface of the sample close to the WSL was carefully ground using SiC-based abrasive papers of the following grit sizes: P800, P1200 (Siawat 1913, Sia Abrasives, Frauenfeld, Switzerland), P2000, P2500 (Smirdex, Lefki–Xanthi, Greece). Running water was used as lubricant. During grinding the sample was thinned in such a manner that the plane of interest crossed the internal portion of the WSL. Following grinding, polishing was conducted with diamond oil-based suspensions (MetaDi, Buehler, Lake Bluff, IL, USA) with particles of 6 and 1 μm diameters. The lubricants GreenLube (Allied, Rancho Dominguez, CA, USA) and BlueLube (Benchmade, Oregon City, OR, USA) were used respectively. The final polishing was made using a sol-gel suspension (MasterPrep, Buehler, Lake Bluff, IL, USA) based on alumina 0.05 μm diameter particles with distilled water as a lubricant. All suspensions were applied with a soft, synthetic, woven, no-nap cloth (Trident, Buehler, Lake Bluff, IL, USA). Each grinding and polishing step was followed by ultrasonic cleaning of the sample in distilled water (Sonorex RK 31, Bandelin, Berlin, Germany) for 3 min.

Using an optical Greenough stereomicroscope (Stemi 305, Zeiss, Shanghai, China) with a colour video camera (Axiocam 105, Zeiss, Oberkochen, Germany) in reflected light images of the tooth crown, including the four areas under study, were made.

The sample was investigated using a wall mounted dental X-ray device (Xelium Ultra, Swidella International Group Limited, Guangdong, China) in order to qualitatively assess the mineral density variation in the WSL area. The voltage on the X-ray tube was 70 kV ± 10%, current 8 mA ± 20%. The nominal focal spot diameter was 0.8 mm. During the experiment, three images were taken with different exposure times: 0.3 s, 0.4 s, 0.5 s.

Determination of mineral density of the sample was conducted using a micro-CT device (SkyScan 1176, Bruker, Kontich, Belgium). A tooth slice in an Eppendorf-type test tube and calibration phantoms in cylindrical tubes (both filled with distilled water) were placed on the semicylindrical bed adapter. Calibration phantoms were represented by a pair of rods composed of epoxy resin with embedded fine calcium hydroxyapatite powder at concentrations of 0.25 and 0.75 g/cm^3^. These phantoms were used for constructing a linear relationship between grey level and mineral density, that allowed conversion of the measured grey value of the tooth slice to an estimated mineral density [35,36]. The micro-CT research was divided into two parts: the first one was conducted using Cu+Al filter and the second one with the Al filter. Within each of the parts, all four tooth areas under study were examined. For the first micro-CT study, a Cu (thickness 0.04 mm) +Al (thickness 0.5 mm) filter of the X-ray beam was used. The following scanning parameters were applied: X-ray tube voltage 80 kV, current 300 µA, pixel size 8.87 μm, sample rotation at each step 0.3°, X-ray tube rotation of 180°, exposure time 1.275 s, ring artefacts correction and Gaussian filter were applied. In the process of scanning, 657 projections for both the tooth slice and the phantoms were obtained using specialized software (CTvox, Bruker, Kontich, Belgium). For the second micro-CT study, a Cu filter (thickness 0.1 mm) of the X-ray beam was used. The following scanning parameters were applied: X-ray tube voltage 90 kV, current 270 µA, pixel size 8.87 μm, sample rotation at each step 0.3°, X-ray tube rotation on 180°, exposure time 1.45 s, ring artefacts correction and Gaussian filter were applied. We generated 657 projections for both the tooth slice and the phantoms.

For the same four tooth areas the mechanical properties were evaluated using a nanoindentation device (NanoTest 600 Platform 3, Micro Materials, Wrexham, UK). The experiments were carried out at a constant temperature of 27.0 ± 0.1 °C in a closed chamber, a calibrated diamond Berkovich indenter was used. The following load profile was applied: the load linearly increased for 20 s, held constant for 30 s, then linearly decreased for 20 s. The thermal drift was recorded and corrected using the device software. The typical and maximum values of the calculated thermal drift rate were 0.4 nm/s and 2 nm/s, respectively. The maximum load $P_{max}$ for all the experiments was 50 mN. To prevent shrinkage of the tooth structure the sample was maintained wet with saline droplets using a syringe pump (Terufusion TE–332, Terumo, Leuven, Belgium). The droplets were applied on the sample between indentations in order to prevent the influence of the droplet impact on the indenter. Values of the reduced Young’s modulus $E_{r}$ and indentation hardness H for each of the areas were obtained using the Oliver–Pharr method [37]. During the entire experiment, the indenter displacement h was recorded while the load P was applied. The maximum value of the displacement (attained at $P_{max}$) was denoted as $h_{max}$. Thus, the output of each experiment was a load–displacement curve. Holding load at its maximum value for a time before unloading eliminated the effect of any creep response on the unloading “branch”. The unloading “branch” was approximated by the function

(1) $$P=a{\left(h-h_{r}\right)}^{m},$$

where a and m are fitting parameters, $h_{r}$ is the residual depth of the imprint, i.e., the amount of displacement of the indenter at which the material ceased to resist during unloading. Using this approximation, the derivative called indentation stiffness *S* was determined at $h_{max}$:

(2) $$S=\frac{dP}{dh}\left(h_{max}\right),$$

To evaluate the reduced Young’s modulus the following formula was used:

(3) $$E_{r}=\frac{S\sqrt{\pi }}{2\beta \sqrt{A_{c}}},$$

where $A_{c}$ is the projected contact area (the projection of the contact surface on a plane orthogonal to the axis of indentation), β is a correction factor. For triangular contact area it is considered to be 1.034 [38]. Indentation hardness was then calculated using the following formula:

(4) $$H=\frac{P_{max}}{A_{c}},$$

The nanoindentation research was divided into two parts. In the first part for each tooth area under study 12 identical indentations were performed with 100 µm column and row offset (a four-by-three position matrix was used) and the results were averaged. In the second part a 10 × 20 matrix of identical indentations were made with the same offset in the area, entirely covering the WSL, the enamel around it, the adjacent dentine–enamel junction and associated dentine bordering the enamel WSL region. This matrix was used for the construction of the reduced Young’s modulus and indentation hardness maps.

For the surface topography research of the four areas under study an AFM (Nano Compact, Phywe, Göttingen, Germany) was used. The surface scanning was conducted in dynamic mode. The device was equipped with monocrystalline Si probe with an Al coating, a resonance frequency of 190 ± 60 kHz and cantilever stiffness of 48 N/m. Scanning velocity was 0.3 ms per line. The resolution along the axis was 1.1 nm. The quadratic mean noise level in the dynamic mode across the z axis (height) was 0.5 nm.

The Raman spectra were measured using a He-Ne laser (wavelength of the laser excitation was 633 nm) in the Raman spectrometer (inVia Reflex, Renishaw, Wotton-under-Edge, UK) with an Edge filter. The backscattering scheme with a Leica optical microscope was used (the resolution of the spectra was less than 0.5 cm^−1^). The laser beam diameter on the sample was of 1–2 μm. The Raman spectra measured were corrected using the Bose–Einstein temperature factor.

For the final part of the research a Crossbeam 340 (Zeiss, Oberkochen, Germany) SEM was used. Prior to the study the tooth was ultrasonically cleaned for 7 min and dehydrated according to the protocol proposed by Bertassoni and Swain [39]. This protocol included immersing the tooth slice in reagent grade acetone solutions of 25, 50, and 70% (*v/v*) for 5 min each, followed by 80, 90, 95, and 100% (*v/v*) for 15 min each, and finally by two consecutive immersions in 100% (*v/v*) acetone for 30 min each. After that, the tooth slice was immersed in hexamethyldisilazane (HMDS) overnight which was permitted to evaporate under a gas ventilation hood. As a volatile organosilicon compound HMDS enables further dehydratation of the organic matrix of the tooth. Additionally, after the HMDS treatment the sample was held in the vacuum chamber for ~ 1.5 h at the pressure 2 × 10^−4^ mbar. The research was conducted using the Everhart–Thornley secondary electron detector with an acceleration voltage of 1 and 2 kV.

## 3. Results

### 3.1. Optical Observations

**Figure 1:**
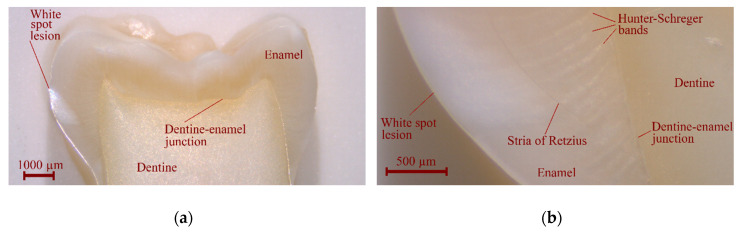
Optical microscopy of the sample after final polishing: (**a**) the crown overview; (**b**) the white spot lesion (WSL) area.

Figure 1 shows the cross-section of the tooth crown through the WSL along with the surrounding enamel, dentine–enamel junction and dentine.

The Hunter–Schreger bands as well as the stria of Retzius are clearly visible, which testifies to the quality of the sample polishing. The images clearly show the absence of cracks on the surface of the sample, which indicates the carious origin of the lesion (not the consequence of pressing of a dental instrument onto the surface of the tooth during extraction). The borders of the WSL inside the enamel are reasonably sharply defined. The dentine bordering the WSL and the sound dentine area on the opposite medial side of the tooth are visually indistinguishable from each other.

### 3.2. Bitewing X-ray

**Figure 2:**
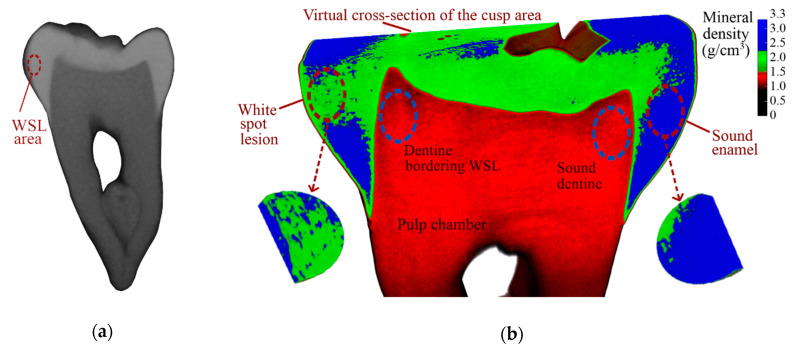
Mineral density research of the sample: (**a**) bitewing X-Ray image (exposure 0.5 s); (**b**) micro-CT (Cu + Al filter) with virtual tablets for the demineralized area of WSL and the sound enamel. Each tablet is cross-sectioned for better visualization of the mineral density by depth. Dentine bordering the WSL and sound dentine is marked with blue dashed ovals.

None of the obtained images demonstrated an observable decrease in the mineral density of the WSL nor in the bordering dentine area. The typical image obtained with the exposure time 0.5 s is shown in Figure 2a (the WSL area is marked with the burgundy dotted line).

### 3.3. Micro-CT

Using software (Nrecon, Bruker, Kontich, Belgium), the projections taken with both Al + Cu and Cu filters were reconstructed into sets of virtual tooth sections. Usage of the calibration phantoms and determining the X-ray attenuation coefficient according to the Bruker recommendations for a mineralized material (such as bone, dentine, and enamel) made it possible to construct a map of the mineral density across the sets of the virtual tooth sections (Figure 2b).

For the WSL area a cylindrical shape region of surface was chosen in the software (CTan, Bruker, Kontich, Belgium) in such a way to cover the main demineralization region in width and length without affecting the bordering enamel. After that a number of projections, where signs of demineralization were visualised, were chosen in such way to cover the main demineralization region in depth without affecting the underlying sound enamel. Thus, a virtual tablet of the WSL area was formed (Figure 2b, left bottom). The same tablets were then constructed for the other three areas of interest for both filters. Results of the measurements of mineral density are summarized in Table 1.

### 3.4. Nanoindentation

**Figure 3:**
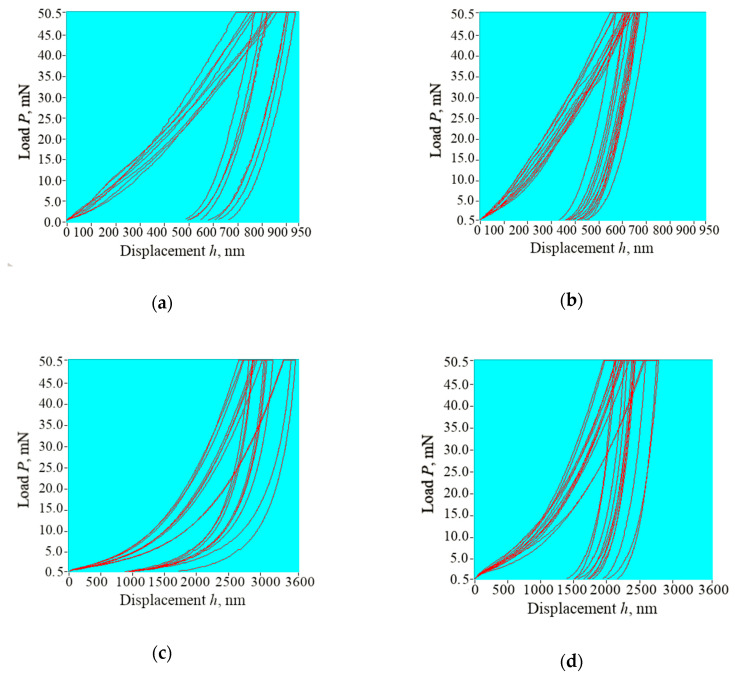
Load-displacement curves for the tooth areas: (**a**) WSL; (**b**) area of sound enamel on the opposite medial side of the tooth; (**c**) dentine bordering the WSL; (**d**) dentine bordering the area of sound enamel. Note the very dissimilar loading and unloading curves for the dentine bordering the WSL in (**c**).

Figure 3 shows the load–displacement curves for each of the tooth areas under study obtained in the first part of the nanoindentation experiments. Each curve contains loading and unloading “branches”, and a horizontal segment produced during the holding period at maximum load. For both carious areas the maximum value of the displacement *h_max_* is greater than for the sound counterparts. The character of the load–displacement curves for enamel WSL and sound enamel areas are similar. Comparing the curves for the dentine areas, we observed an obvious shape change of the unloading “branch” for the dentine bordering the WSL. In addition, indentation creep while holding *P_max_* constant was recorded for each curve. Determined values of mechanical properties and indentation creep (average and standard deviation) are summarized in Table 2.

**Figure 4:**
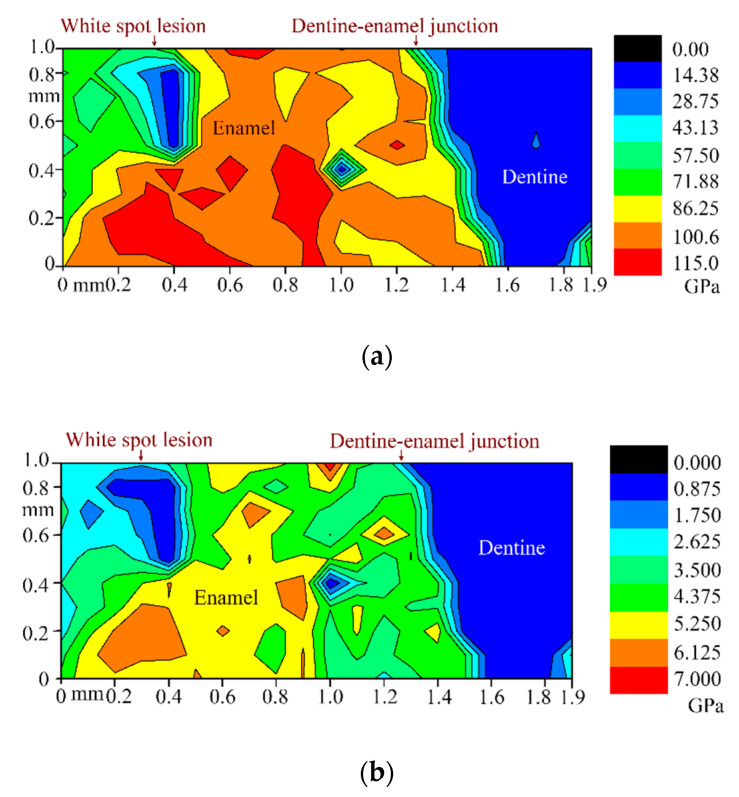
Maps of the mechanical characteristics of WSL and surrounding tooth areas: (**a**) reduced Young’s modulus; (**b**) indentation hardness.

Figure 4 demonstrates the results of the second part of the nanoindentation experiments: the maps showing distribution of the reduced Young’s modulus and indentation hardness in the area partly covering the WSL and extending to the dentine bordering the WSL. It is seen that the properties of the enamel bordering the WSL are reduced despite its appearance as being sound based on optical images.

### 3.5. Atomic Force Microscopy

**Figure 5:**
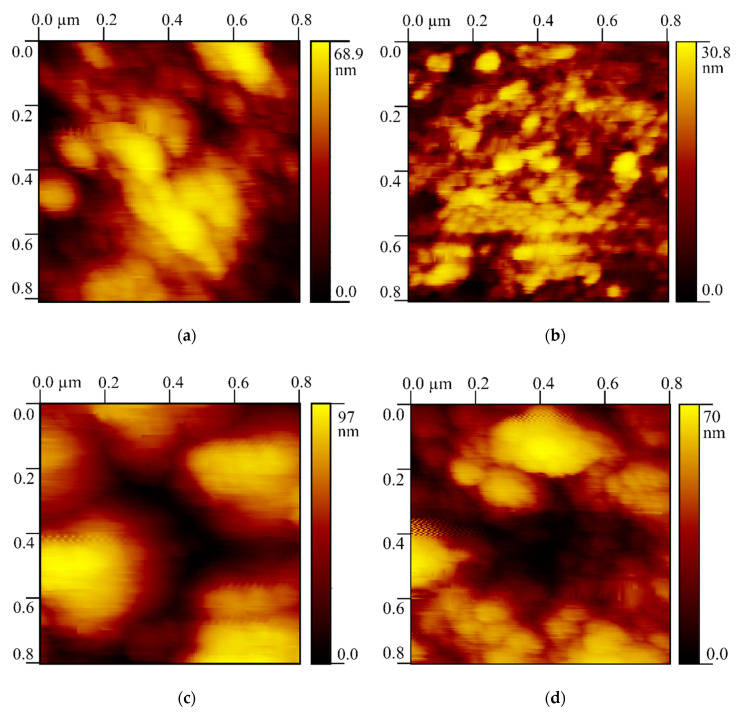
Surface topography of the tooth areas: (**a**) natural enamel WSL; (**b**) area of sound enamel on the opposite medial side of the tooth; (**c**) dentine bordering the WSL; (**d**) dentine bordering the area of sound enamel.

For each of the four areas under study a series of AFM images were taken. Figure 5 shows one image for each of the areas. These images were chosen as they represent the most common features at the nanoscale that we observed among each of the areas. For each image, a 0.8 × 0.8 µm scanning field was chosen for the convenience of visual comparison of the topographies of the areas under study. For comparing the areas affected by caries with their sound counterparts the average surface roughness *R_a_* for each of the images was measured using software (Gwyddion, Czech Metrology Institute, Brno, Czech Republic). Due to the irregularities observed on the visualized surface we considered measurement of the roughness based on a single direction as not being completely indicative. 

Thus, average roughness *R_a_* was measured in three directions: horizontal, vertical and diagonal. In each of the directions five profiles were constructed. After that the average value (of the 15 profiles) with the standard deviation was calculated. Maximum roughness height was denoted as *R_t_*. The measurement results for the images in Figure 5 are presented in Table 3. 

According to the Student’s *t*-test for dependent (paired) samples the difference between the average *R_a_* values measured for carious and sound pairs of enamel and dentine was found to be significant. The value of *t*-test was significantly higher than the threshold chosen for statistical significance α = 0.05 and 14 degrees of freedom (2.145). In the pair of enamel WSL and sound enamel the *t*-test value was 7.889. In the pair of dentine bordering WSL and its counterpart, the *t*-test value was 3.884. 

### 3.6. Raman Spectroscopy

**Figure 6:**
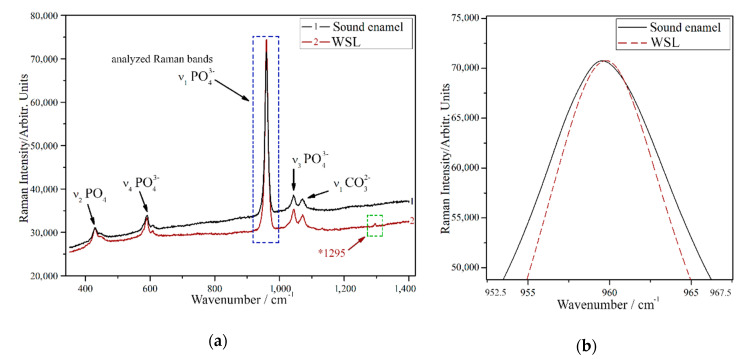
Results of the Raman spectroscopy research: (**a**) spectra sound enamel and WSL (average of four spectra each); (**b**) the shift of the most intense band position due to the carious process.

Typical Raman spectra of human sound and carious enamel obtained upon excitation with a 633 nm laser beam are shown in Figure 6a. The rising background visible in the spectra appeared due to fluorescence emission. The Raman bands of enamel observed were related to the phosphate (PO_4_^3−^) and carbonate (CO_3_^2−^) groups, which play the leading role in the structure of hydroxyapatite crystallites. The most intense band was found at 959 cm^−1^ (ν_1_ PO_4_^3−^), it is marked with the blue dotted rectangular on Figure 6a. Other bands associated with the ν_2_ and ν_4_ PO_4_^3−^ vibrations were detected in regions from 390 cm^−1^ to 490 cm^−1^ and from 560 cm^−1^ to 625 cm^−1^, respectively. The bands corresponding to ν_3_ PO_4_^3−^ were visible in the region from 1010 cm^−1^ to 1060 cm^−1^, whereas the band assigned to CO_3_^2−^ groups is detected near 1071 cm^−1^. The shift of the most intense band as well as its full width at half maximum (FWHM) were calculated for the most intense ν_1_ PO_4_^3−^ band. For showing any band shift, the Raman bands are presented in Figure 6b, which was normalized and the background was subtracted manually from each raw spectrum using a polynomial curve. The values of the ν_1_ PO_4_^3−^ band in case of sound enamel was 959.5 cm^−1^ similar to observations of Ko et al. [20]. This value shifted to 960.5 cm^−1^ for the WSL area. FWHM of sound enamel was 10.85 cm^−1^. This value reduced to 10.59 cm^−1^ for the WSL area. Besides, on the Raman spectra of WSL we observed a small band at 1295 cm^−1^, not visible for the sound enamel (marked with the green dotted rectangular on Figure 6a).

### 3.7. Scanning Electron Microscopy

**Figure 7:**
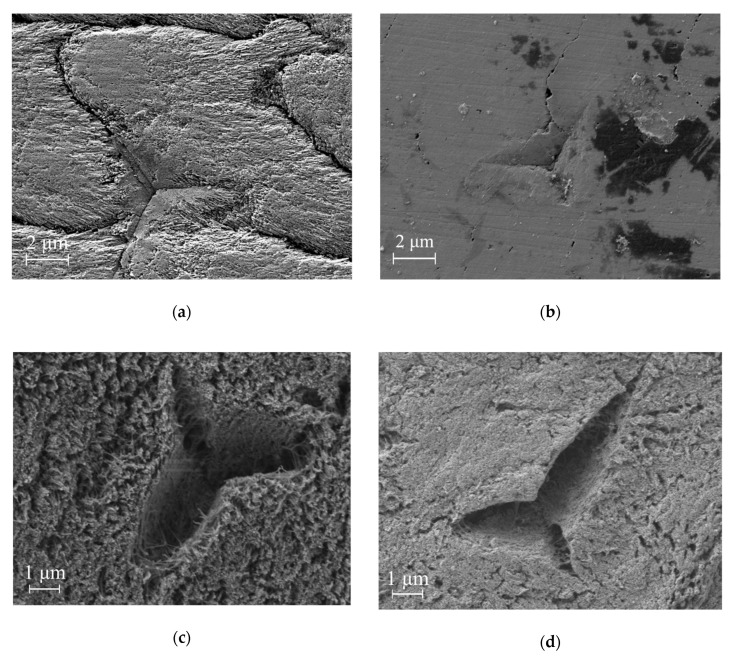
Surface and indenter impressions on the various tooth areas: (**a**) WSL, (**b**) area of sound enamel on the opposite medial side of the tooth, (**c**) dentine bordering the WSL, (**d**) dentine bordering the area of sound enamel. Note that for the sound areas study we rotated the sample 180º.

Figure 7 demonstrates the indentation impressions as well as the polished surface nearby for the tooth areas under study. For each area one impression was chosen for the SEM research. The WSL area (Figure 7a) revealed a loss of structure about the interrod areas suggesting that demineralization had weakened this region much more than in the core of the rod structure (the borders of the enamel rods can be clearly distinguished on the figure). The core of the rod structure was weakened as well, although not so severely. The sound enamel shows minimal relief at the rod interface, the surface is smooth and covered with a thin smear layer [40] (Figure 7b). Visual observation of the impressions in dentine (Figure 7c,d) suggests that severe dehydration has occurred in the SEM because of the low vacuum.

## 4. Discussion

Each of the experimental methods in the present work reveals differences between the carious and sound areas of the tooth. Comparing the mineral density derived from micro-CT for these areas, we observed the following: using Al+Cu filter, the WSL showed a small reduction in mineral density compared to sound enamel counterpart (up to 5.2% lower), while dentine areas demonstrated the same values of mineral density. In the case of a Cu filter, the enamel WSL showed practically the same level of reduction in mineral density (up to 4.9% lower), dentine bordering WSL showed also a small reduction in mineral density compared to sound dentine counterpart (up to 1.5% lower). The difference in enamel density is relatively small compared to those found by Huang et al. in [19], where the median value of the relative difference in mineral density reached 28% (2.9 g/cm^3^ in sound area and 2.1 g/cm^3^ in WSL). It may indicate that the WSL in the studied sample was at an earlier stage of its development. 

Reduction in mechanical properties of WSL enamel was found and compared to its sound counterpart: $E_{r}$ was up to 38.1% lower and H up to 42.5% lower. Values of elastic modulus for sound enamel fitted in the range 47–120 GPa found in literature [22,41,42,43,44,45]. Compared to the findings of Huang et al. [19,22], the values of elastic modulus for the WSL were greater, which also may be due to the earlier stage of the WSL development. During caries development, the crystallites become partially demineralized generally at the cores and some interfaces as observed during transmission electron microscopic study [46], where the central dark line inside a crystallite represented a stacking fault resembling a dislocation and residual stresses were present about the core. This line possesses a higher concentration of Mg and Na [16] and crystallographic point defects (impurities, vacancies etc.) that would contribute to it being more soluble resulting in greater porosity. It is thus not surprising in this regard that we observed an increase of creep for the enamel WSL area during nanoindentation experiments (up to 69.3% higher, Table 2). Mapping of the mechanical properties revealed a reduction for the enamel adjacent to the WSL (although not as severe as within a WSL). This fact can be important for the selection of area for early caries treatment by a clinician. It should be noted that both indentation hardness and reduced Young’s modulus of the inner region of WSL located in the proximity of the dentine–enamel junction are close to those for the dentine bordering the WSL.

Dentine bordering a WSL also demonstrated a decrease in the mechanical properties: $E_{r}$ was up to 55.0% lower and H up to 35.3% lower (despite the fact that the reduction in mineral density of dentine bordering the WSL was barely visible as well as on the optical and bitewing X-Ray images it also did not differ from the sound counterpart). The shape of the unloading “branch” of load–displacement curves for this partially demineralized dentine (Figure 3c) differs from the same “branch” for the sound dentine (Figure 3d), which indicates a change of the mechanism of resistance to loads caused by the mineral loss. A similar effect was reported by Angker et al. [47] and considered to be associated with the greater porosity and permeability of this structure. This leads to an increase in the water content and the breakdown of organic component [48]. It is interesting, however, that these processes did not influence the indentation creep, for which, for dentine bordering a WSL, the data lay within the standard deviation of the sound dentine.

AFM results revealed statistical significance of the *R_a_* changes caused by caries development (at a significance level of α = 0.05). This change appears to be higher for enamel than for dentine. The increase in the surface microgeometrical parameters for the carious areas is consistent with the SEM observations, where the same areas appear to be less structurally sound. We observe that both caries-affected areas demonstrate some gaps between the crystallites and even deep grooves, especially at the interrod region. For the WSL enamel, this most likely occurs because of the ability of the interrod enamel to allow diffusion of acid produced by bacteria along this region more readily than for the bulk regions of the rods. This is not surprising as the interrod structure possesses a higher organic content compared to the enamel rods [43] and the crystallites in the interrod regions appear to deviate up to 90º from those of the rod core [49], plus they are less densely packed [50,51]. The microstructure of dentine bordering a WSL is far more open compared to the sound counterpart because of the loss of mineral especially within the collagen-rich intertubular region. In the absence of cavitation and biofilm formation on dentine, the collagen fibers are well preserved by the dehydration protocol. We note the rounding of the indentation induced crack tips in the demineralized dentine versus the sharper cracks in the sound dentine. This is associated with the lower stiffness of the demineralized dentine as the crack shape is related to the stress intensity factor and the modulus of the material. The dehydration of the sample has caused embrittlement of the collagen and the indentation impressions to act as stress concentrations sites where crack initiation occurs (Figure 7d) rather than being simply compressed as for moist dentine. 

We should also bear in mind the susceptibility of more fragile partially dissolved crystallites in enamel to breaking up while polishing and forming the smear layer. The bigger aggregates of hydroxyapatite crystallites on the dentine areas (Figure 5c,d) were most likely formed during sample polishing as well and are not related to the caries disease.

The Raman bands of sound enamel observed are in good agreement with those in the literature [41,52,53]. The shift of the most intense band position to a higher wavenumber (Figure 6b) is in a good agreement with the results of Buchwald et al. [54]. This shift along with the change of the FWHM is a sign of enamel structure relaxing during demineralization as the most strained central line area of the hydroxyapatite during caries dissolves, resulting in less strain in the remaining “skeleton” of the crystallite. The shape of the phosphate-type band ν_4_ PO_4_^3−^ stretching from 560 cm^−1^ to 625 cm^−1^ appears to be similar for WSL and sound enamel. This observation slightly differs from the one made by Natarjan et al. [55], where flattening was observed for the similar band in carious enamel. We suggest that this difference may be attributed to the more severe stage of caries studied in [55], namely a brown spot lesion (BSL). 

The observed band at 1295 cm^−1^ coincides with the amide III δ (=CH) band found in bone [56]. As no such band was found on the sound enamel covered with the remains of the smear layer after grinding and polishing procedures, it may be related to the caries disease. In sound enamel, the protein-rich regions can be found as small clusters between the crystallites [57] in such a way that the protein (presumably amelogenin, that contains amide III group [58] is naturally distributed across the surface of the crystallites. On the other hand, the grooves and gaps between the crystallites separated from each other as a result of crystallites dissolution (in addition to breakdown of some of them during polishing) visualized using AFM may have acted as sites for collecting water and organic material [59] including proteins. Besides, the porous structure of the weakened carious enamel causes more scatter of the Raman laser beam, which generates more signal overall. This phenomena is in a good agreement with [55], where opening of the enamel structure after acid treatment of BSL enabled the infiltrated resin to be detected on Raman spectra (gaps formed by acid on the place of damaged crystallites contributed to the increasing of the signal). This increased volume of the proteins becomes sufficient for the Raman spectroscope to detect an amide group in the carious part of the sample. Previously the amide III band present in Raman spectra was reported to be a sign for a carious disease by Timchenko et al. [60], however that research was conducted at a later and more advanced stage of caries (BSL).

The observed features using Raman spectroscopy are barely noticeable compared to the resolution of the device: the shift for the most intense band was 1.0 cm^-1^, the change in its FWHM was 0.26 cm^-1^, the amide band is the least intense among the others present in the spectra. On the other hand, all these features were stable and repeatable on the current sample for all four measurements of carious and sound enamel on the tooth slice. In order to understand the significance of these results for the non-invasive in vivo detection of early caries, various methods already proposed [61,62,63,64] and further experiments on the external surfaces of teeth should be conducted. 

## 5. Conclusions

The work reveals some fundamental changes emerging inside human enamel and dentine at the first clinically visible stage of the carious disease from several points of view. The complex of characteristics—mineral density, reduced Young’s modulus and hardness, and average roughness—was found for the natural enamel WSL and dentine bordering it. The properties obtained were compared to those of sound counterparts of the aforementioned areas on the same tooth. The results were supplemented by bitewing X-ray, optical, scanning electron microscopy and Raman spectroscopy observations. The significant reduction of the mechanical properties was recorded for both carious areas accompanied by the abnormality of the unloading response and change of the character of indentation marks caused by mineral loss. Increase of indentation creep for WSL area was recorded. The maps of mechanical properties show that the enamel outside the WSL is weakened despite its appearance as being sound on optical and bitewing X-ray images. At the same time, the decrease in mineral density of the WSL area and bordering dentine was rather small. The average roughness of polished surfaces was considerably increased in the caries-affected areas caused by the demineralization process, and the surface relief changed as well. Raman spectroscopy research resulted in detection of interesting repeatable features connected to changes in the molecular composition in the carious areas (the most intense PO_4_^3−^ band shift, the change in FWHM and a new band appearance, detected and described for the WSL), which need additional specific studies for possible further implementation in dental practice as caries detection tools.

## Figures and Tables

**Table 1:** Mineral density of the tooth areas.

Filter	Group	Area	Mineral Density, g/cm^3^	Standard Deviation, g/cm^3^
Al+Cu	WSL	Enamel	2.21	0.06
		Dentine	1.21	0.06
	Sound	Enamel	2.33	0.08
		Dentine	1.21	0.03
Cu	WSL	Enamel	2.35	0.07
		Dentine	1.29	0.05
	Sound	Enamel	2.47	0.08
		Dentine	1.31	0.06

**Table 2:** Mechanical properties of the tooth areas.

Group	Area	Reduced Young’s Modulus *E_r_*, GPa	Indentation Hardness *H*, GPa	Indentation Creep, nm
WSL	Enamel	69.12 ± 4.97	2.79 ± 0.46	64.60 ± 18.00
	Dentine	6.04 ± 0.78	0.22 ± 0.04	155.22 ± 23.24
Sound	Enamel	111.57 ± 8.95	4.85 ± 0.62	38.16 ± 9.92
	Dentine	13.41 ± 1.55	0.34 ± 0.06	175.96 ± 41.20

**Table 3:** Surface roughness of the tooth areas.

Tooth Area	Average *R_a_* by 15 Profiles, nm	Standard Deviation, nm	*R_t_*, nm
Natural enamel WSL	8.9	3.6	68.9
Sound enamel	2.8	1.0	30.8
Dentine bordering WSL	14.2	4.2	97.0
Sound dentine	7.3	3.9	70.0
